# Early-onset smoking theory of compulsivity development: a neurocognitive model for the development of compulsive tobacco smoking

**DOI:** 10.3389/fpsyt.2023.1209277

**Published:** 2023-07-13

**Authors:** Aldo Alberto Conti, Alexander Mario Baldacchino

**Affiliations:** ^1^Department of Child and Adolescent Psychiatry, King's College London, Institute of Psychiatry, Psychology and Neuroscience, London, United Kingdom; ^2^Division of Population and Behavioral Science, University of St Andrews School of Medicine, St Andrews, United Kingdom

**Keywords:** compulsive tobacco smoking, early-onset smokers, adolescents, anterior insular cortex, neuroimaging, nicotine addiction

## Abstract

According to the literature, individuals who start tobacco smoking during adolescence are at greater risk of developing severe tobacco addiction and heavier smoking behavior in comparison with individuals who uptake tobacco smoking during subsequent developmental stages. As suggested by animal models, this may be related to the unique neuroadaptive and neurotoxic effects of nicotine on adolescents' fronto-striatal brain regions modulating cognitive control and impulsivity. Previous research has proposed that these neuroadaptive and neurotoxic effects may cause a heightened reward-oriented impulsive behavior that may foster smoking relapses during quit attempts. However, developments in the field of addiction neuroscience have proposed drug addiction to represent a type of compulsive behavior characterized by the persistent use of a particular drug despite evident adverse consequences. One brain region that has received increased attention in recent years and that has been proposed to play a central role in modulating such compulsive drug-seeking and using behavior is the insular cortex. Lesion studies have shown that structural damages in the insular cortex may disrupt smoking behavior, while neuroimaging studies reported lower gray matter volume in the anterior insular cortex of chronic smokers compared with non-smokers, in addition to correlations between gray matter volume in the anterior insular cortex and measures of compulsive cigarette smoking. Based on the findings of our recent study reporting on early-onset smokers (mean age at regular smoking initiation = 13.2 years) displaying lower gray matter and white matter volume in the anterior insular cortex compared to late-onset smokers (mean age at regular smoking initiation = 18.0 years), we propose that the anterior insular cortex may play a central role in mediating the association between smoking uptake during adolescence and smoking heaviness/tobacco addiction during adulthood.

## Introduction

Tobacco smoking is the leading cause of preventable deaths worldwide. According to the WHO, approximately 8 million individuals die each year as the direct and indirect consequences of chronic tobacco smoking (1). Tobacco cigarettes contain approximately 7,000 toxic chemicals that are responsible for chronic and lethal cardiopulmonary diseases such as obstructive pulmonary disease (OPD), strokes, lung cancers, throat cancers, and coronary heart disease (CHD). Tobacco cigarettes also contain nicotine which is a highly addictive psychoactive substance as it activates nicotinic acetylcholine receptors (nAChRs) and stimulates the release of the neurotransmitter dopamine (DA) in the brain's meso-cortico-limbic reward pathway, thereby causing a powerful reinforcing effect contributing to the vicious cycle of addiction.

Individuals with tobacco addiction usually start this maladaptive behavior during adolescence (before 21 years of age) (2). Epidemiological studies revealed a negative relationship between early smoking initiation during adolescence, smoking chronicity, and severe nicotine dependence during adulthood (3–5). A genetic study conducted by Kendler et al. (6) on monozygotic twin pairs showed siblings who started smoking at a mean age of 14.6 years display more severe nicotine dependence and more intense craving for tobacco during adulthood compared with siblings who started smoking at a mean age of 19.1 years (6). Notably, animal models have shown that the adolescent brain is more susceptible to the addictive and neurotoxic properties of nicotine in comparison with the adult brain as it may influence the expression of genes involved in the neuroplasticity of frontal brain regions and may cause changes in macromolecular constituents indicative of cell loss (reduced DNA) and altered cell size (protein/DNA ratio) in the cerebral cortex, among other neurostructural and neurochemical alterations (7–10).

In line with the above evidence, Debry and Tiffany (11) proposed the tobacco-induced neurotoxicity theory of adolescent cognitive development (TINACD). According to the TINACD, the earlier the age of smoking initiation is, the more severe the damage of fronto-striatal developing brain regions (e.g., prefrontal cortex, PFC and anterior cingulate cortex, ACC) that are important for self-control and emotion regulation is. This may result in poor modulation of reward-driven responses, therefore rendering early-onset smokers (smoking initiation at < 16 years) more prone to smoking relapses during quitting attempts in comparison with late-onset smokers (smoking initiation at ≥ 16 years). This impulsive behavior has been partially supported by neuropsychological studies that reported adult early-onset smokers (mean age at regular smoking initiation = 13.2 years) to present poorer response inhibition in comparison with late-onset smokers (mean age at regular smoking initiation=17.7 years) (12).

However, in the last decade, several scholars in the field of addiction neuroscience have proposed drug addiction (including tobacco addiction) to represent a type of compulsive behavior that is characterized by a tendency toward repetitive, habitual actions, repeated despite adverse consequences (13). For instance, the symptoms listed in DSM-V for “substance use disorder” (SUD) reflect a compulsive behavior, which is characterized by excessive time spent searching for the drug of abuse when it is not available, by the prioritization of the search for the drug of abuse over other activities (e.g., familial, social, and work-related), by a failure to avoid self-harm, and by the craving for the substance of abuse (14). The inability to inhibit urges toward drug-related rewards in tobacco smokers while they are experiencing negative emotional states (and that according to the TINACD may be related to the neurotoxic effect of tobacco on the adolescent brain) can only be considered a neurocognitive facet of tobacco addiction, and it does not necessarily explain the compulsive smoking behavior that a majority of adult chronic smokers show.

According to Figee et al. (14), the compulsive drug-taking behavior manifested by individuals with addiction overlaps (from a neurocognitive point of view) with the compulsive behavior manifested by individuals affected by obsessive–compulsive disorder (OCD). A review conducted by the same authors showed that the compulsive behavior manifested by individuals affected by addictions and by those affected by OCD is characterized by (a) impaired reward processing associated with blunted responses in the ventral striatum to non-drug-related rewards (for individuals with drug addiction) and non-symptom provoking stimuli (for individuals affected by OCD), (b) negative reinforcement (compulsive behavior to avoid anxiety and stress) associated with abnormalities in brain anti-reward and anxiety circuits involving the bed nucleus of the stria terminalis (BST), amygdala, and habenula, (c) cognitive inflexibility associated with structural and functional abnormalities in the orbitofrontal cortex (OFC), and (d) insensitivity toward punishment associated with decreased neuronal activity in the medial-prefrontal cortex (mPFC)–ventrolateral striatum circuitry and insular cortex. In support of this paradigm, a recent meta-analysis conducted by Stevens et al. (15) on 44 VBM studies reported that 736 individuals affected by alcohol use disorder and 995 individuals affected by obsessive–compulsive disorder (OCD) share a low GM volume in the right insular cortex, while an activation likelihood estimation (ALE) meta-analysis conducted by Klugah-Brown et al. (16) on 144 fMRI studies reported shared neurofunctional alterations in the anterior insular cortex between 2,428 individuals affected by SUDs (e.g., cocaine, alcohol, and tobacco), 361 individuals affected by internet gaming disorder (IGD), and 715 individuals affected by OCD.

The insular cortex (or Island of Reil) is a brain region that has received increased attention in the last decades and has been increasingly recognized as pivotal in the development of compulsive drug use in the field of addiction neuroscience, albeit its mechanisms are still largely unknown. From a neuroanatomical point of view, the insular cortex can be roughly divided into anterior and posterior insula subregions. As stated by Namkung et al. (17), “*each subregion has different cytoarchitectonic features, connectivity, and therefore functions”*. Particularly, functional connectivity studies have shown the posterior insula to be connected with brain regions modulating somatosensory and motor processing including parietal, occipital, and temporal cortices (17, 18), and the anterior insula to be connected with fronto-cortical brain regions (e.g., prefrontal cortex, PFC; anterior cingulate cortex, ACC), the ventral striatum, and the amygdala. The anterior insular cortex is considered instrumental in integrating interoceptive information projected by the posterior insula with emotional, cognitive, and motivational functions (17). A recent review conducted by Molnar-Szakacs and Uddin (19) proposed the anterior insular cortex as a “*gatekeeper of executive control*” as it integrates and prioritizes internal and external stimuli to guide and maintain adaptive behaviors. For instance, the anterior insular cortex can be considered a key node of the salience network (SN), and neuroimaging studies have shown that the anterior insula modulates the switching from the internally oriented default mode network (DMN) to the externally oriented executive control network (CEN) (19). The right dorsal anterior insular cortex plays a central role in this regard by “*acting as a causal outflow hub at the junction of these large-scale brain networks*” (19). Indeed, fMRI studies have shown a causal effect of the right anterior insular cortex in activating the CEN and deactivating the DMN across task paradigms and stimulus modalities (20).

Therefore, it is plausible to think that functional and structural disruptions of the anterior insular cortex (particularly of the right anterior insular cortex) may impair the interoceptive awareness of individuals with and their capacity to switch their attention toward more adaptive external stimuli; that is, they may continue to engage in compulsive drug taking as they may be less aware of the negative physiological effects of a particular drug [i.e. self-administration in the face of punishment or aversive consequences as proposed by Figee et al. (14)]. An fMRI study conducted by Wang et al. (21) provided evidence for the role of the anterior insular cortex in modulating interoceptive awareness by showing an association between interoceptive attention (as measured by a breathing detection task) and anterior insular cortex activation in 44 healthy adult participants. Furthermore, individuals who suffered lesions in the anterior insular cortex showed disrupted interoceptive discrimination accuracy and sensitivity.

Intriguingly, a study conducted by Somnez et al. (22) reported that individuals with heroin, alcohol, and cannabinoid addiction display less interoceptive awareness (as measured by a heart tracking task) compared to controls with no drug addiction. Structural neuroimaging studies have instead shown lower gray matter (GM) volume in the anterior insular cortex of individuals with addiction compared to controls with no drug addiction and/or occasional drug users. The majority of these studies also reported statistical correlations between reduced GM volume in the anterior insular cortex, addiction severity, and/or compulsive drug taking. For example, Weng et al. (23) reported GM atrophy in the bilateral anterior insular cortex of adolescents affected by gaming addiction with a mean age of 16.2 years in comparison with matched controls with no gaming addiction. GM atrophy in the bilateral anterior insular cortex correlated with greater addiction severity as measured using the Young's Internet Addiction Scale (YIAS) (23). An MRI study conducted by Grodin et al. (24) reported low GM volume and thickness in the bilateral anterior insular cortex of 60 individuals with alcohol addiction compared to 49 controls with no alcohol addiction. GM volume and thickness in the anterior insula correlated negatively with scores on the ‘Obsessive Compulsive Drinking Scale' (OCDS). The evidence is also compelling if we consider chronic tobacco smoking. For instance, Stoeckel et al. (25) reported lower GM volume in the left anterior insular cortex of chronic smokers compared with non-smoker controls. GM volume of the anterior insular cortex correlated with a greater number of cigarettes smoked daily. Similarly, Wang et al. (26) showed GM volume in the bilateral anterior insular cortex of chronic smokers to be negatively correlated with the severity of nicotine addiction (FTND scores). Morales et al. (27) reported negative correlations between GM volume in the right anterior insular cortex, pack years, and scores on the Cigarette Dependence Scale (CDS) in young cigarette smokers (mean age=19 years). As stated by the authors “*The CDS assesses an individual's subjective experience of symptoms such as craving, compulsion to use, levels of stress when unable to smoke, and difficulty quitting or controlling intake”* (27).

Animal models have also shown an effect of structural and functional alterations of the anterior insular cortex on compulsive behavior and drug intake. For example, Belin-Rauscent et al. (28), who defined the insula as “*a neurobiological gate for the development of compulsive behavior*” reported a correlation between lower thinness in the bilateral anterior insular cortex and heightened motor impulsivity (as assessed by a five-choice serial reaction time task, 5-CSRTT) and compulsive water intake (as assessed by a schedule-induced polydipsia task, SIP) in 140 rats that underwent a neurosurgical and neuroimaging procedure (28). Furthermore, surgical lesions of the bilateral anterior insular cortex disrupted the manifestation of both impulsive and compulsive behaviors in rodents (28). Another study (29) conducted on rodents using a foot-shock-punished cocaine self-administration procedure reported rats with compulsive cocaine intake (punishment-resistant state) to present increased neural activity in the anterior insular cortex compared with non-compulsive rats. Jadhav and colleagues (30) reported adolescent rats to be more persistent in lever pressing during a reward (saccharine)-delivery foot-shock administration procedure compared with adult rats. As stated by the authors, “*lever presses decreased with increased shock intensity from 0.22 to 0.33 mA in all rats, but to a significantly lesser extent in adolescents. Strikingly, the adolescents persisted in lever pressing despite the 0.22 mA mild electrical foot shock, suggesting a compulsive-like reward-seeking behavior”* (30). The same authors also observed a decrease in mRNA expression of the zif268 protein, as well as a lower excitability of L5 pyramidal neurons, and a weaker glutamatergic synaptic input to the anterior insular cortex of adolescent rats showing compulsive saccharine intake (30).

Overall, the above evidence suggests that the anterior insular cortex is a key region mediating compulsive drug-taking (including tobacco smoking). By also considering the strong association between smoking initiation during adolescence and severe tobacco addiction during adulthood (3–6), we raise the question of whether the anterior insular cortex (among other biopsychosocial factors that are outside the scope of this manuscript) may play a pivotal role in mediating this association.

## Structural differences in the insular cortex between early-onset smokers and late-onset smokers

Recently, we conducted a VBM study hypothesizing that 11 adult smokers (mean age at recruitment = 25.2 years) who started regular tobacco use at 13.2 years of age (i.e., early-onset smokers) would present heightened impulsive and risky choices, in addition to reduced GM and WM volume in frontal brain regions modulating such behaviors in comparison with 17 adult smokers (mean age at recruitment= 30 years) who started regular smoking at 18 years of age (i.e., late-onset smokers) and 24 matched non-smoker controls (31). Our hypotheses were based on the previously cited TINACD theory (11). However, our findings contradicted such hypotheses as early-onset smokers did not display heightened impulsive and risky choices (as assessed by the five-choice delay-discounting task and by the Cambridge Gambling Task, respectively) in comparison with late-onset smokers. Early-onset smokers displayed higher risk-taking behavior only when compared to non-smoker controls (for more detailed results please refer to our previous study 31).

**Figure 1:**
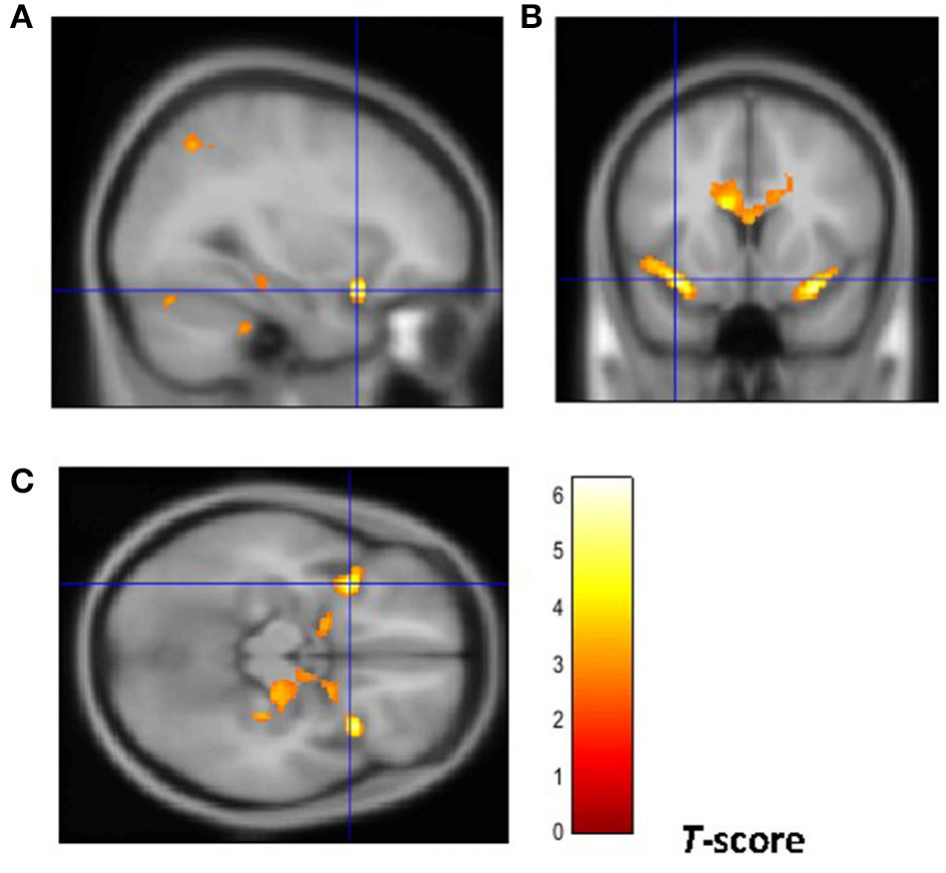
Brain regions of interest displaying lower GM volume in early onset smokers in comparison to late onset smokers. The figure shows early onset smokers to display lower GM volume in the bilateral anterior insular cortex [region of interest centered at 32, 15, −18 MNI coordinates in sagittal plane **(A)**, and at −34, 14, −15 MNI coordinates in coronal **(B)** and axial **(C)** planes] in comparison to late onset smokers. The cluster forming threshold consisted in *p* < 0.01 with a minimum of 100 contiguous voxels per cluster at a whole-brain corrected level. TIV, age, and biological sex were inserted as covariates of no interest.

**Figure 2:**
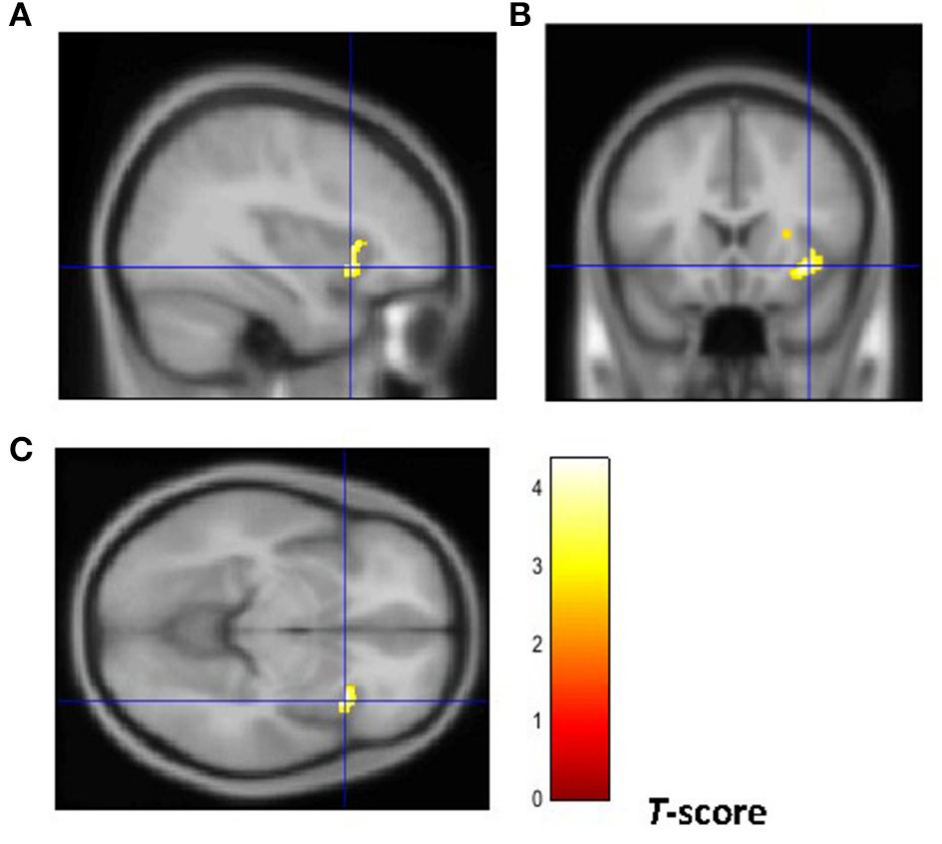
Brain regions of interest displaying lower WM volume in early onset smokers in comparison to late onset smokers. The figure shows early onset smokers to display lower WM volume in the right anterior insular cortex [region of interest centered at 36, 18, −8 MNI coordinates in sagittal **(A)**, coronal **(B)**, and axial **(C)** planes] in comparison to late onset smokers. The cluster forming threshold consisted in *p* < 0.01 with a minimum of 100 contiguous voxels per cluster at a whole-brain corrected level. TIV, age, and biological sex were inserted as covariates of no interest.

From a neuroanatomical point of view, early-onset smokers displayed lower GM volume in the right ACC and lower WM volume in the left anterior corpus callosum, left anterior insular cortex, and bilateral thalamus in comparison with non-smokers (31). Remarkably, early-onset tobacco smokers displayed significantly lower GM volume in the bilateral anterior insular cortex and lower WM volume in the right anterior insular cortex in comparison with late-onset smokers (31) (Figures 1, 2).

It is worth noticing that early-onset smokers and late-onset tobacco smokers were well-matched in relation to tobacco use/exposure characteristics (i.e. no. of cigarettes smoked per day, pack-years). Early-onset tobacco smokers displayed higher scores on the FTND in comparison with late-onset smokers albeit this difference did not reach statistical significance. No correlations were identified between tobacco use/exposure characteristics and GM/WM volume of early-onset smokers' anterior insular cortex. As reported in our previous study, this may indicate that such structural abnormalities were not due to differences in tobacco exposure between the two groups (31).

**Figure 3:**
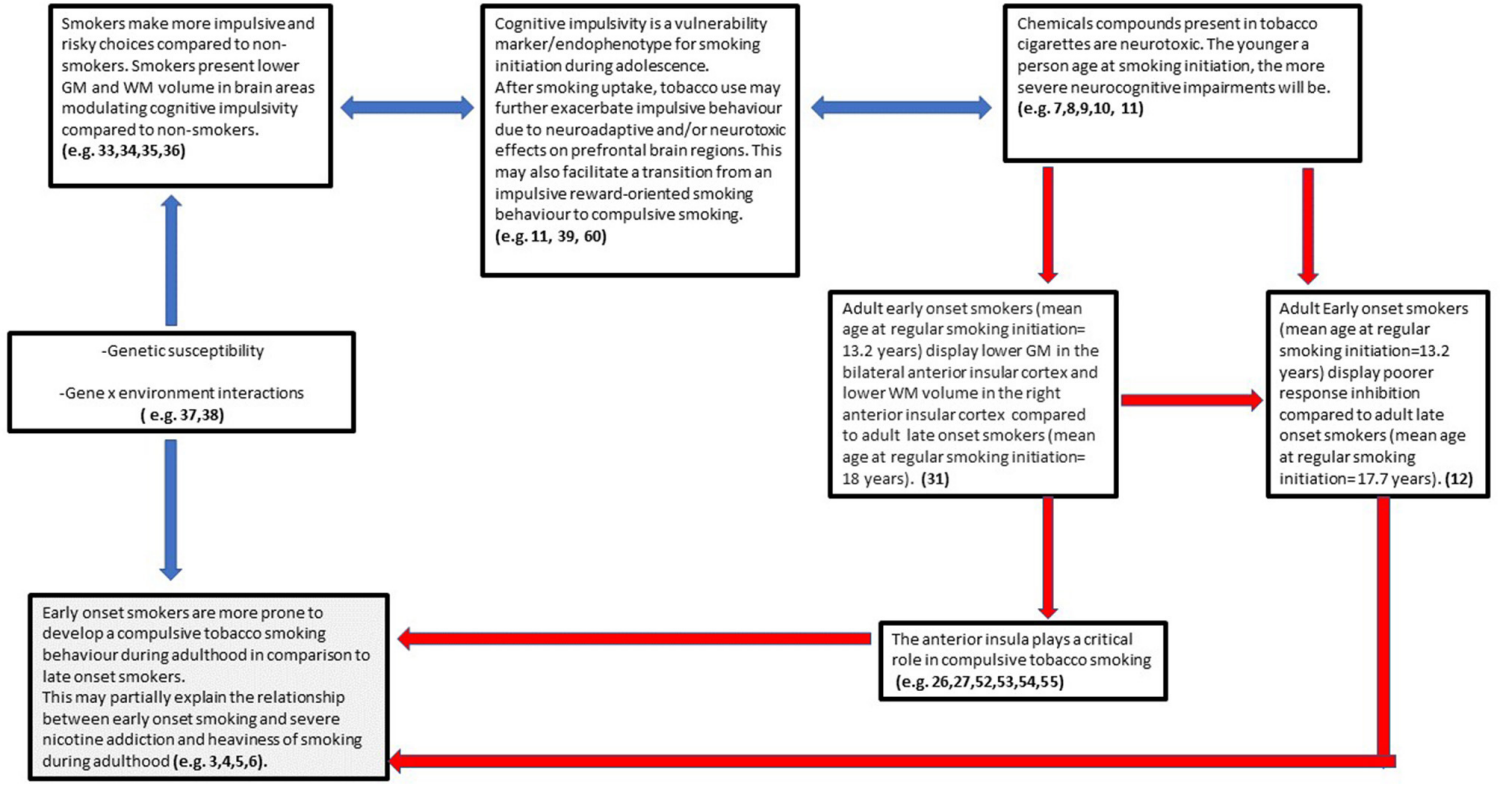
Early onset smoking theory of compulsive smoking development (EASCD). Red arrows indicate links that need to be tested by future research.

Considering the findings from the above study, in addition to the evidence pertaining to the pivotal role of the anterior insular cortex in mediating tobacco addiction and compulsive drug use, we have developed a theoretical model with the aim to motivate future research investigating the relationship between smoking initiation during adolescence and tobacco addiction/compulsive smoking during adulthood under a neurocognitive point of view (Figure 3).

## Early-onset smoking theory of compulsive smoking development (EASCD)

The postulation of the above model (Figure 3) encompasses findings from the last decade in the field of addiction neuroscience and is based on the assumption that cognitive impulsivity/reward-based decision-making and compulsivity can be considered two different neurocognitive constructs, with cognitive impulsivity being more relevant for drug use initiation and compulsivity for drug use maintenance/chronicity (32). Indeed, a wealth of studies has shown that chronic tobacco smokers present heightened impulsive choices (high delay discounting rates) in comparison with non-smokers [e.g., (33–36)]. However, despite animal models reporting nicotine to cause neurostructural and neurochemical alterations in frontal brain structures modulating impulsivity (7–10), there is a paucity of evidence about a possible causal neurotoxic effect of tobacco on such structures in humans. Genetic research has instead shown that the preference for smaller and sooner rewards over larger delayed rewards in tobacco smokers (including adolescents) is highly heritable and studies have proposed choice impulsivity/delay discounting to be a pre-existing endophenotype conferring vulnerability to smoking initiation (37–39).

Mechanistically, the transition from an impulsive “reward-oriented” smoking behavior to compulsive smoking may be facilitated by the neurostructural and neurochemical alterations caused by the chronic consumption of tobacco on relevant brain regions (e.g., anterior insular cortex) that are still developing during adolescence. Indeed, a neuroimaging study conducted by Dennis et al. (40) showed that the density of 15 fiber connections between the insula and the frontal and parietal cortex decreased from 12 to 30 years old in a sample of 307 young people. According to the authors, this decrease in fiber density may represent the neuro-maturational processes of myelination and synaptic pruning that peak during adolescence. Nevertheless, as our VBM study (31) employed a cross-sectional design, it is not possible to infer causality, and the possibility that structural brain abnormalities in the anterior insular cortex of early-onset smokers were pre-existing cannot be excluded. Regardless of a possible neurotoxic effect of tobacco on the adolescent brain, it could be postulated that structural brain abnormalities in the anterior insular cortex of early-onset smokers may facilitate a compulsive smoking behavior and therefore heaviness of smoking during adulthood. This may partially explain the relationship between early smoking initiation during adolescence and severe tobacco addiction identified by the previously cited epidemiological and twin studies (3–6).

As introduced previously, there is compelling evidence suggesting that structural and functional brain abnormalities in the anterior insular cortex may facilitate the development of a compulsive smoking behavior characterized by a loss in interoceptive awareness. This view has been also supported by neuroimaging studies showing reduced activation in the insular cortex of individuals with addiction compared with controls with no drug addiction while responding to positive and negative (non-drug related) interoceptive stimuli during risk-processing tasks (41–45). Indeed, as stated by Stewart et al. “*addiction may reflect a discrepancy between an individual's predicted vs. actual internal state known as the bodily prediction error, an imbalance that could in turn adversely influence the degree of future drug-related approach vs. avoidance behavior”* (45). As the anterior insular cortex processes and integrates somatosensory feeling states in order to guide future decisions, it has been proposed that reduced anterior insular cortex activation to negative stimuli may induce individuals with drug addiction to not process negative consequences of drug use as prediction errors to correct and optimize future behavior (45, 46). This may be particularly relevant for the right anterior insular cortex as it modulates the switching from the DMN to the CEN (19, 20). Notably, our VBM study has shown early-onset smokers to display GM volume reductions in the bilateral anterior insular cortex in comparison with late-onset smokers. Importantly, lower WM volume was only identified in the right anterior insular cortex of early-onset smokers but not in the left anterior insular cortex. This may suggest that the right anterior insular cortex may be particularly affected by smoking uptake during early adolescence and, intuitively, it may be particularly relevant in modulating a possible compulsive smoking behavior.

According to Robbins and Everitt (47), the loss in interoceptive awareness may decrease the flexibility of drug users when anticipating or experiencing an aversive event related to the use of the drug and may be linked to the transition from a reward-oriented to a compulsive drug-taking behavior; that is, early-onset smokers may be less interoceptively aware of the unpleasant physiological effects of tobacco smoking (e.g., shortness of breath, chest tightness, increased heart rate, unpleasant smell of tobacco combustion), and therefore, they may be more prone to engage in tobacco smoking and less motivated to “switch” their attention toward more adaptive behaviors (e.g., smoking cessation) in comparison with late-onset smokers.

Poor response inhibition could also be considered a neurocognitive facet of this hypothesized compulsive smoking behavior as impairments in response inhibition are a predominant feature of psychiatric disorders characterized by high levels of compulsivity (e.g., OCD) (48). Indeed, neuroimaging studies have shown the anterior insular cortex to also modulate the initiation and suppression of actions. For example, a meta-analysis conducted by Swick, Ashley, and Turken (49) on fMRI and PET studies employing both Go/No Go and stop-signal tasks revealed the bilateral anterior insular cortex to be activated during stop-signal tasks, and the right anterior insular cortex to be activated mainly during Go/No Go tasks, while a recent fMRI study conducted by Tomiyama et al. (50) revealed functional connectivity between the supplementary motor area and the bilateral anterior insular cortex in OCD patients to be correlated with poor response inhibition (as assessed by a stop-signal task) in comparison with healthy controls. Intriguingly, the study conducted by Masshhoon et al. (12) reported about 10 early-onset smokers displaying poorer response inhibition in comparison with 10 late-onset smokers as assessed by a Go/No Go task (12). However, the relationship between poor response inhibition and functional and/or structural disruptions of the anterior insular cortex is yet to be explored in early-onset tobacco smokers.

A desensitized interoceptive system may not be the only neurobiological process through which the anterior insular cortex may render early-onset smokers more prone to develop a compulsive smoking behavior in comparison with late-onset smokers. An alternative view is that lower GM and/or WM volume in smokers' insular cortex “*could actually reflect a more sensitized (or overactive) interoceptive insular cortex system*” (51). For instance, several fMRI studies reported an overactivation of the anterior insular cortex in chronic smokers while they were exposed to smoking cues (52–54). The overactivation of the anterior insular cortex to smoking cues has been also associated with tobacco cravings (55). According to Naqvi et al. (55), chronic tobacco smoking may create a bodily state of “perturbed” homeostasis that may refer to the emergence of withdrawal symptoms (e.g., anxiety, irritability) when an individual ceases to smoke tobacco. For a chronic smoker who is suffering withdrawal symptoms, smoking cues may represent a possibility to return the body to a “controlled” homeostatic state. When an individual is exposed to smoking cues during tobacco withdrawal, the insula generates a prediction-error signal representing the discrepancy between the current perturbed homeostatic state and the predicted controlled homeostatic state that may be achieved by smoking. Ultimately, this discrepancy between the actual and predicted bodily homeostatic state induces craving and motivates the smoker to relapse (55). An overactive insular cortex system that is instrumental in modulating tobacco cravings may also support the findings of several lesion studies that found individuals who sustained traumatic injuries in the insular cortex to experience a marked reduction in cigarette urges, withdrawal symptoms and undergo a disruption of their tobacco smoking behavior, in comparison with patients who sustained injuries in other brain areas (56–58). However, it should be pointed out that one study did not find any associations between right or left insular cortex lesions in stroke patients and smoking status at 3 months follow-up after hospitalization (59). This may imply that other biopsychosocial factors that vary between individuals (e.g., intention to quit smoking) may mediate the association between lesions in the insular cortex lesions and smoking cessation.

The deactivation of the anterior insular cortex, which has been associated with deficits in interoceptive awareness and consequently to a punishment-resistant compulsive smoking behavior, and the overactivation of the anterior insular cortex to smoking cues, which has been associated with tobacco cravings, may not be mutually exclusive in early-onset smokers. As proposed by Luscher et al. (60), there is a differentiation under a neurobehavioural point of view between drug seeking and drug taking. By reviewing animal models of intravenous drug administration, the authors proposed that_*punishment-resistant instrumental taking responses* can be considered a predominant feature of compulsive drug taking in rats. However, compulsive drug seeking mainly occurs while rats are exposed to cue-conditioned responses during drug abstinence. Specifically, “*cue-controlled seeking after abstinence has revealed the phenomenon called the ‘incubation of craving', whereby a drug-conditioned reinforcer supports seeking behavior that progressively increases the longer the period of abstinence following long-access self-administration sessions”* (60). Therefore, considering the previously discussed evidence about the role of the anterior insular cortex in modulating both punishment-resistant drug intake and tobacco cravings, it could be postulated that structural abnormalities in the anterior insula may render early-onset smokers more prone to develop both a compulsive tobacco-seeking and a tobacco smoking behavior in comparison with late-onset smokers.

## Limitations, future directions, and concluding remarks

The model proposed in this study is highly speculative and intuitive at this stage as in our VBM paper we did not investigate for differences between early-onset smokers and late-onset smokers regarding a compulsive tobacco-seeking and smoking behavior. Furthermore, we did not investigate possible differences between the two groups of participants in relation to anterior insular deactivation (and its possible association with a loss of interoceptive awareness) or overactivation to smoking cues (and its possible role in modulating tobacco cravings). The sample recruited in our VBM study was also small (31) and may not be representative of the early-onset smoking population.

Additionally, scholars have recently proposed that the persistence of drug use despite adverse consequences (and that according to our theoretical paradigm may be associated with a decrease in interoceptive awareness) is not a unitary construct and may encompass three dissociable cognitive, motivational, and behavioral pathways (61). For instance, McNally et al. (61) have proposed that the persistence of drug use despite adverse consequences/punishment may be characterized by (1) a cognitive pathway for recognition of adverse consequences, (2) a motivational pathway for valuation of these consequences, and (3) a behavioral pathway for responding to these adverse consequences. As stated by the authors, “*These pathways are dissociable but they are neither mutually exclusive nor exhaustive. They may operate dynamically within the same individual at different times*” (61). Other scholars have instead criticized the systematic use of the term “compulsivity” to characterize persistent drug use despite (mild) punishment in animal models of addiction-like behavior without ruling out alternative accounts (62).

Nonetheless, considering the strong association between the structural and functional abnormalities in the anterior insular cortex and what we have defined as a “compulsive” tobacco smoking behavior (in line with the previously cited preclinical, neuroimaging, and lesion studies) and by also considering the correlation between early-onset smoking and heaviness of tobacco smoking during adulthood (3–6), future research should aim at investigating the theoretical model proposed in this paper by:

1. Replicating our structural neuroimaging findings by recruiting a larger sample size and by utilizing neurocognitive tasks assessing the multifaceted nature of compulsivity (an example could be the Intra-External Dimensional Shift task, which is a computerized test designed to assess for cognitive inflexibility), response inhibition, interoceptive awareness, and the compulsive smoking behavior of participants by utilizing measures such as the CDS. These studies should also investigate possible differences in interoceptive awareness between early-onset smokers and late-onset smokers and may correlate GM/WM volume in the anterior insular cortex of early-onset smokers with measures of interoceptive awareness, scores of neurocognitive tasks, and measures of compulsive smoking behavior.
2. Conducting fMRI studies exploring whether early-onset smokers present decreased activation in the anterior insular cortex during risk-processing tasks while responding to negative and positive interoceptive stimuli in comparison with late-onset smokers.
3. Conducting fMRI studies exploring whether early-onset smokers present greater activation in the anterior insular cortex while exposed to smoking cues during tobacco withdrawal in comparison with late-onset smokers and correlate such activation with measures of tobacco craving (e.g., Brief Questionnaire of Smoking Urges).

Findings from these studies may confirm whether early-onset smokers present a more compulsive smoking behavior in comparison with late-onset smokers during adulthood and may verify whether this compulsive smoking behavior is associated with structural and/or functional abnormalities in the anterior insular cortex. These results may inform the development of targeted brain stimulation treatments (e.g., transcranial magnetic stimulation, transcranial direct stimulation) aiming at preventing the transition from a reward-directed impulsive smoking behavior to compulsive tobacco-seeking and smoking in early-onset smokers.

## Data availability statement

The raw data supporting the conclusions of this article will be made available by the authors, without undue reservation.

## Ethics statement

The studies involving human participants were reviewed and approved by the London Bromley Research Ethics Committee (REC) (REC Reference Number: 19/LO/1176) and by the University of St. Andrews Teaching and Research Ethics Committee (UTREC) (UTREC Approval Code: MD14516). The patients/participants provided their written informed consent to participate in this study.

## Author contributions

AC was responsible for the conceptualization of the content and write-up of the current manuscript. AB provided supervision. Both authors critically reviewed the content and approved the final version for publication.
